# Type 1 diabetes-associated cognitive impairment and diabetic peripheral neuropathy in Chinese adults: results from a prospective cross-sectional study

**DOI:** 10.1186/s12902-019-0359-2

**Published:** 2019-03-27

**Authors:** Xin Ding, Chen Fang, Xiang Li, Yong-Jun Cao, Qi-Lin Zhang, Yun Huang, Jian Pan, Xia Zhang

**Affiliations:** 1 Division of Neonatology, Children’s Hospital of Soochow University, Suzhou, 215025 China; 2 Department of Endocrinology, the Second Affiliated Hospital of Soochow University, Suzhou, 215004 China; 3 Department of Neurology and Suzhou Clinical Research Center of Neurological Disease, the Second Affiliated Hospital of Soochow University, 1055 Sanxiang Road, Suzhou, 215004 China; 4 Institute of Pediatric Research, Children’s Hospital of Soochow University, Suzhou, 215025 China

**Keywords:** Type 1 diabetes mellitus, Cognitive function, MoCA, Diabetic periphery neuropathy, C peptide

## Abstract

**Background:**

To compare neurocognitive functioning of Type 1 diabetic mellitus (T1DM) and healthy adults, and explore risk factors of cognitive dysfunction of T1DM patients, especially the association between cognitive impairment and diabetic peripheral neuropathy (DPN).

**Methods:**

Seventy T1DM (age: 32.17 ± 9.57 yr., duration: 8.99 ± 7.02 yr) patients and 48 healthy volunteers were included. All subjects received evaluation of MMSE and MoCA scales. Cognitive function of T1DM patients was evaluated in different cognitive domains. Risk factors affecting cognitive function were further explored.

**Results:**

Three patients with educational level ≤ 6-year were excluded from final analysis. Scores of both MMSE (28.4 ± 1.7 vs. 29.1 ± 1.0, *P* = 0.005) and MoCA scales (25.9 ± 2.7 vs.27.1 ± 2.4, *P* = 0.017) in T1DM group were lower than that in control group. For MMSE scale, scores of orientation (9.60 ± 0.79 vs.9.87 ± 0.39, *P* < 0.001) and language function (8.56 ± 0.65 vs.8.83 ± 0.38, *P* < 0.001) in T1DM groups were lower than that in control group. For MoCA scale, scores of attention and concentration (2.30 ± 0.74 vs.2.57 ± 0.58, *P* < 0.001), visuospatial/executive function (4.32 ± 0.91 vs.4.64 ± 0.63, *P* < 0.001), memory (2.96 ± 1.50 vs.3.66 ± 1.28, *P* < 0.001), language function (5.71 ± 0.69 vs.5.87 ± 0.39, *P* = 0.007), and abstraction (1.55 ± 0.68 vs.1.82 ± 0.42, *P* < 0.001) were lower in T1DM group than that in control group. Logistic regression showed age, fasting C peptide, educational level and nerve conduction velocity (NCV) were associated with cognitive dysfunction diagnosed by MoCA scores for the patients with type 1 diabetes.

**Conclusions:**

T1DM adults had mild to moderate cognitive impairment, mainly presenting as dysfunctions of attention and concentration, visuospatial/executive, language, and abstraction. In addition to age, fasting C peptide level, and educational level, DPN, as a diabetic complication, was identified to be associated with cognitive impairments.

**Electronic supplementary material:**

The online version of this article (10.1186/s12902-019-0359-2) contains supplementary material, which is available to authorized users.

## Background

T1DM is a chronic autoimmune disease affecting multiple organs, which is characterized by total insulin deficiency essential for glucose metabolism. This in turn can lead to impairments in the brain and can affect cognitive function. People with T1DM have been shown to have mild to moderate cognitive impairment as measured by neuropsychological testing compared to non-diabetic controls [1]. T1DM patients were reported to have cognitive dysfunction in different cognitive domains of varied degrees, ranging from general intellectual testing, to specific deficits with visuospatial abilities, motor speed, writing, attention, reading, and psychomotor efficiency [2, 3]. However, results have not always been consistent, regarding the extent and domains of cognitive difficulties and their associated risk factors.

Diabetic peripheral neuropathy (DPN) is also a common, debilitating, and distressing complication affecting up to 30–50% of patients with diabetes [4]. DPN has only been considered as a disease of the peripheral nervous system in the past. Nevertheless, with advances in noninvasive magnetic resonance imaging (MRI), more and more evidences have shown central nervous system (CNS) involvement in DPN [5, 6]. Therefore it is speculated that cognitive impairment may be associated with DPN in T1DM.

In this study, we first compared the neurocognitive functioning of T1DM adults and healthy adults. In T1DM adults, we then examined the association between the cognitive function and multiple factors. We first confirmed that age, education and C peptide level are associated with the cognitive impairment in T1DM patients as previously reported [7, 8]. Next, using nerve conduction velocity (NCV) as an indicator of DPN, we investigated the relationship between cognitive impairment and occurrence of DPN in a Chinese population. Our findings thereby shed light on studies of effects of DPN on cognitive functions in T1DM patients.

## Methods

### Study participants

We conducted a cross-sectional study of 118 adults (70 cases with T1DM and 48 healthy controls without chronic illness) older than 18-yr from the endocrine department and medical examination center at the Second Affiliated Hospital of Soochow University from Jan 2013 to Oct 2014. The diagnosis of type 1 diabetes mellitus was accorded with diagnostic criteria of WHO in 1999, made by endocrine specialists. T1DM patients fulfilled three criteria: duration of diabetes at least six months, right-handed, and stable glycemic control (HbA1c < 11%). Most patients in our study present in the classic way: rapid onset of hyperglycemia, weight loss, with marked symptoms. All patients have completed tests of pancreatic function, immune markers and islet autoantibodies before diagnosis of T1DM by more than 2 endocrine specialists and were treated using insulin with the guidance of endocrine specialists. Before enrollment, two endocrine and neuropsychological specialists confirmed that all subjects were confirmed that they were suitable for having neuropsychological assessments and no hospitalization within 3 months. HbA1c was done on the day of cognitive assessment. All subjects have been screened for potential causes of cognitive dysfunction. Before they were enrolled into the study, all of them were screened with routine blood test, electrolytes tests and thyroid function to exclude diseases that could potentially cause cognitive decline. In addition, we carefully reviewed all past medical records before we accepted subjects into this study. Exclusion criteria included: other neurological diseases, presence of nondiabetic neuropathies, history of long-term alcohol consumption, recurrent severe hypoglycemia, hypoglycemic unawareness, severe hepatic and renal dysfunction, incoordination for examination (severe anxiety or depression, mental retardation, language disorders). In the same period, demographically similar healthy controls who received a complete check of body health status at our health examination center, were consecutively enrolled. In the healthy examination center, as persons like, they can have biochemical examination of blood such as serum lipids, hepatic and renal function, and fasting glucose, also including tumor markers, thyroid function and so on by the same detecting instruments as T1DM patients. Additionally patients can have a CT or X- ray scan of any part of the body as indicated, even including MRI examinations. There are transcranial and carotid dopplers. Psychological assessments are included too. A matching ratio of 1:2 was used to enroll healthy controls and T1DM patients. Healthy controls were confirmed by normal fasting plasma glucose (FPG) and HbA1c levels, with matched age and educational levels to diabetic patients. For each healthy control included in the study, a standard diagnostic procedure was given including biochemical examinations of blood such as serum lipids, hepatic and renal function, glucose tests, tumor markers, and thyroid function as well as cranial CT scan and detailed history inquiry to exclude those with hypothyroidism, DM, tumor, hepatic and renal diseases, neurological illness, or peripheral neuropathy. More details were referenced to our previous publication [9]. The study was approved by the local Ethics Committee of the Second Affiliated Hospital of Soochow University, and all patients or their family members signed informed consent forms.

### Clinical variables and outcome evaluation

Detailed demographics information, including age, sex, duration, height, weight, educational levels, history of severe hypoglycemia, hyperglycemia, diabetic ketoacidosis (DKA) and family history of diabetes, was collected. HbA1c, FPG, blood lipids [total cholesterol (TC), triglyceride (TG), high density lipoprotein (HDL), low density lipoprotein (LDL)], serum uric acid, serum C peptide, urine microalbumin, and urine creatinine of fasting blood and urine samples were analyzed.

### Diagnosis of DPN

The diagnosis of DPN was made using ≥1 abnormal attribute in ≥2 separate nerves by NCV, adapted from the Toronto consensus. The cut off points for NCV measures are listed in the (Additional file 1: Tables S1-5). Briefly, if the conduction velocity is lower than − 20%, or amplitude is lower than lower limit, or latent period is longer than + 20%, or amplitude is lower than lower limit, the attribute is considered as abnormal. Bilateral comparison is also important. Significant differences between two sides means one side may be abnormal. One experienced specialist without knowledge of the history of subjects did the examinations in our study. We now included detailed protocol for NCV measure.

All patients received nerve conduction studies the same way described in our published article [9]. In our previous study, CPT results showed left sural nerves were most easily injured [9]. Therefore NCV of left sural nerves were used as a continual variable in the regression analysis of association between cognitive function and DPN.

### Assessment of cognition

All participants (patients and controls) underwent assessment by Mini Mental State Examination (MMSE) and Montreal Cognitive Assessment (MoCA). MMSE is a 30-point questionnaire that is applied extensively in clinical and research settings to measure cognitive impairment. The test examines functions including registration (repeating named prompts), attention and calculation, recall, language, ability to follow simple commands and orientation [10]. MoCA is a more elaborate test of a one-page 30-point test assessing several cognitive domains including memory recall, visuospatial abilities, attention and concentration, language, abstraction, calculation, and orientation [11]. The interpretations of MMSE and MoCA scores may need to be corrected for educational attainment. 1 point should be added to the total score of MoCA if the person has less than or equal to 12-year education. Any score of MoCA scales greater than or equal to 26 points indicates a normal cognition [11]. To avoid possible influences from particular time/status of the measurement, subjects were asked to complete tests in the morning (10:00 am) after breakfast without coffee and tea. Subjects sat in a quiet room with temperature 24 ± 1 °C, one to one interviewed. All subjects did not take other medicines except insulin. In all enrolled T1DM patients, four were diagnosed as stage I diabetic retinopathy (DR), two as stage II DR, both of which show no overt vision effects. To further confirm this, an ophthalmologist and a neuro-psychologist examined those patients and concluded there was no interference with visuo-spacial assessment for these 6 patients with DR. The evaluation was performed by a qualified neurologist who did not know about the histories of all participants. Before the assessment, all subjects received a fingertip blood glucose test to rule out hypoglycemia and hyperglycemia. The blood glucose levels ranged from 3.9 mmol/L to 10 mmol/L.

### Statistical analysis

Results were expressed as a percentage, median with interquartile ranges, or mean ± SD. Differences in frequencies were compared with the Chi-square test. Comparisons of median values among groups were done by the Student t test, one-way ANOVA, or non-parametric tests, as appropriate. Type 1 patients were categorized into two groups according to the MoCA scores after corrected for educational attainment, a normal cognition group and an abnormal cognition group. Before we did the logistic regression, we examined the co-linearity of data by SPSS. We then applied the stepwise regression to avoid the co-linearity of data. We fitted a logistic model to estimate the association between cognitive function and several risk factors, especially NCV as a continual variable with OR (odds ratio) and 95%CI (confidence interval) after adjusting for potential confounders: sex (female = 0, male = 1), age, fasting glucose levels, serum uric acid concentrations, C peptide, creatinine ratio, HAb1C, educational attainment(no education = 0, ≤6-year education = 1, ~ ≤ 12-year education = 2, > 12-year education = 3),duration, and BMI.

All statistical analyses were 2-sided test. A *P* value< 0.05 was considered statistically significant. SPSS16.0 software (SPSS Inc., IBM, Chicago, IL, USA) was used for all analyses.

## Results

Diabetic and healthy control groups were not significantly different in mean age (*P* = 0.108), sex distribution (*P* = 0.934), and educational attainments (*P* = 0.446) (Table 1). The blood glucose levels ranged from 3.9 mmol/L to 10 mmol/L (mean ± SD: 6.5 ± 2.3 mmol/L) immediately before the cognitive assessment.

**Table 1:** Demographic information of the studied group

	Diabetic group (*n* = 70)	Control group (*n* = 48)	*P* value
Age (mean ± SD,yr)	32.17 ± 9.57	31.56 ± 7.15	0.108
Sex (male no, %)	37 (52.9)	25 (52.1)	0.934
FBG (mean ± SD, mmol/L)	11.33 ± 6.01	4.56 ± 2.03	< 0.001
Duration (mean ± SD, yr)	8.99 ± 7.02	N/A	
Educational attainments (n,%)			
No education = 0	0 (0)	0 (0)	
≤6-year education = 1	2 (2.8)	1 (2.1)	
7 to ≤12-year education = 2	34 (48.6)	29 (60.4)	
> 12-year education = 3	34 (48.6)	18 (37.5)	0.446

### Cognitive function of type 1 patients compared with healthy controls

For educational level was associated with cognitive impairment and there were few patients in educational level ≤ 6-year education group, 3 patients in the group were excluded from the study. Table 2 shows scores from the cognitive assessments for T1DM patients and control subjects. The diabetic duration of T1DM patients is from 0.50 to 37 years, with mean 8.99 ± 7.02 years. The type 1 diabetes group scored lower on both the MMSE and MoCA compared with control subjects (28.4 ± 1.7 vs. 29.1 ± 1.0, *P* = 0.005; 25.9 ± 2.7 vs. 27.1 ± 2.4, *P* = 0.017, respectively). Based on the MoCA scores, there were 26 (38.2%) patients with cognitive impairment in T1DM group and 5 (10.6%) controls in healthy control group, with a significant difference between the two groups (*P* = 0.001).

**Table 2:** Comparison of different cognitive domains of MMSE and MoCA scales between the two groups

	Items	Diabetic group (*n* = 68)	Control group (*n* = 47)	t value	*P* value
MMSE	Orientation	9.60 ± 0.79	9.87 ± 0.39	−4.19	< 0.001
	Memory	2.99 ± 0.10	3.00 ± 0.00	−1.42	0.158
	Attention and calculation	4.70 ± 0.62	4.81 ± 0.53	−1.66	0.098
	Recall	2.56 ± 0.67	2.64 ± 0.60	−1.08	0.279
	Language	8.56 ± 0.65	8.83 ± 0.38	−4.90	< 0.001
MoCA	Attention and concentration	2.30 ± 0.74	2.57 ± 0.58	−3.76	< 0.001
	VA and EF	4.32 ± 0.91	4.64 ± 0.63	−3.78	< 0.001
	Memory	2.96 ± 1.50	3.66 ± 1.28	−4.50	< 0.001
	Language	5.71 ± 0.69	5.87 ± 0.39	−2.71	0.007
	Abstraction	1.55 ± 0.68	1.82 ± 0.42	−4.16	< 0.001
	Calculation	2.88 ± 0.47	2.85 ± 0.41	0.65	0.518
	Orientation	6.00 ± 0.00	5.98 ± 0.14	1.74	0.083

VA and EF means visuospatial abilities and executive functions

### Analysis of specific cognitive domains decline of T1DM patients compared with the healthy controls

For MMSE tests, scores on orientation and language of T1DM patient were significantly lower than those of healthy controls. For MoCA scales, scores on attention and concentration, memory, language, visuospatial and executive abilities, and abstraction of T1DM patients were significantly lower than those of healthy controls (Table 2).

### The correlation between known risk factors and DPN and cognitive impairment of T1DM patients

T1DM patients were divided into two groups according to MoCA scores: impaired cognition group or normal cognition group. We then performed multiple logistic regression analysis. The first categorywas patients’ characteristics: age, gender, educational attainment, BMI, and diabetic duration. Among them, consistent with previous studies [3, 7], age and education showed correlations with cognitive impairment in T1DM patients. We then examined biochemical laboratory variables including fasting glucose, serum uric acid, C peptide, HbA1c, creatinine ratio, in which serum C peptide level was identified as a risk factor for cognitive impairment of T1DM patients. After confirming the accuracy of such logistic regression analysis, we calculated whether the NCV(m/s) showed a correlation with cognitive impairment in T1DM patients. Our results showed that abnormal NCV might be a risk factor for cognitive impairment in persons with Type 1 diabetes (Table 3).

**Table 3:** The association between multiple factors and cognitive impairment in T1DM patients from multivariate analysis

Variables	ß	S.E.	*P*	OR	95% CI
Sex	−0.67	1.04	0.518	0.51	0.07–3.93
Age	0.16	0.07	0.025	1.17	1.02–1.35
FPG	−0.03	0.09	0.726	0.97	0.81–1.16
BMI	0.06	0.09	0.535	1.06	0.88–1.27
Duration	−0.15	0.10	0.123	0.86	0.71–1.05
Education	1.21	1.14	0.019	0.30	0.03–2.80
Uric acid	0.01	0.01	0.062	1.01	1.00–1.02
C peptide	−4.77	2.09	0.023	0.01	0.00–0.51
Creatinine ratio	−0.03	0.03	0.354	0.97	0.90–1.04
HbA1c	−0.29	0.30	0.327	0.74	0.41–1.34
NCV	2.41	1.40	0.046	8.48	1.96–81.34

FPG indicates fasting plasma glucose, *BMI* body mass index, *HBA1C* glycosylated hemoglobin, *NCV* nerve conduction velocity

## Discussion

It has been reported that T1DM patients have mild to moderate cognitive dysfunction compared to non-diabetic controls [12]. The study provides data that will better characterize the impact of T1DM on adult cognitive functions. In addition, we revealed the association between cognitive dysfunction and DPN for the first time in a Chinese population. The results were consistent with the literatures about other races with some difference in affected cognitive domains [13, 14]. In our study, worse memory/language, visuospatial abilities and executive functions, abstraction, attention and concentration performance for T1DM adults were reported, which were basically consistent with previous results [3, 12]. Many of these functions depend on efficient information processing, which is the hallmark of cognitive problem in T1DM patients.

Our analysis revealed that DPN was positively associated with cognitive impairments in T1DM patients. Consistent with this, more and more evidences have been reported on central nervous system involvement for DPN patients [15, 16]. There have been further reports of CNS involvement in DPN including dysfunction of the somatosensory afferent pathways using evoked potentials, thalamic neuronal dysfunction, and perfusion abnormalities seen on MRI studies [17–19]. Selvarajah et al. identified reduced peripheral gray matter volume mainly localized to primary somatosensory cortex and supramarginal gyrus in subjects with established DPN, and greater thalamic gray matter volume reduction in subjects with painless DPN [20]. Clinically relevant white matter hyperintensity (WMH) has been reported to play an important role in cognitive dysfunction and prevalent neuropathies in T1DM patients. Clinically relevant WMH were related to the slower information processing frequently observed in T1DM, and prevalent neuropathies tripled the odds for high WHM burden, independent of age or disease duration [21]. All these data showed a connection between cognitive dysfunction and DPN in T1DM patients.

As complications of the same disease, there might be some similarities in the pathogenic mechanisms of both cognitive dysfunction and DPN. There are also several common risk factors for the development of both cognitive impairment and DPN. For example, the most important one is HAlb1c levels and chronic hyperglycemia state [12, 22]. Hyperglycemia could cause microvascular dysfunction in the blood-brain barrier, autoimmune injury of neurological tissue and alterations in the synthesis, availability, or reuptake of neurotransmitters, such as serotonin. It increases the production of oxygen free radicals and weaken the bodies’ antioxidant ability. Moreover, hyperglycemia inhibits specific Na^+^-dependent carrier and leads to decreased activity of Na^+^-K^+^-ATP enzyme, which results in slowing of nerve conduction velocity. Several studies have reported relationship between neuropsychological test performance and the presence of hyperglycemic complications like neuropathy [3, 23]. The incidence of neuropathy is also associated with some other risk factors, including a raised triglyceride level, body-mass index, smoking, and hypertension. The risk factors for cognitive impairment involve age of onset, and episodes of severe hypoglycemia. It is necessary to conduct clinical studies including all these risk factors to further explore the association between DPN and cognitive dysfunction. To dissect the common pathogenic mechanisms of both, further longitudinal clinical and morphological studies including T1DM patients with both cognitive and DPN are needed.

The current study has some limitations. We cannot determine causality because of the cross-sectional design. The sample size was relatively small. HbA1c levels were only determined once, so the relationship between continued blood sugar levels and cognitive dysfunction has not been discussed. There were seldom DKA or severe hypoglycemia attacks for the subjects, so we have not considered those factors into logistic analysis and the clinical significance of both hypo- and hyperglycemia on cognitive function could not be figured out. Therefore, it is cautioned that our findings should be supported by future work in larger prospective longitudinal studies.

## Conclusions

In conclusion, this study has identified cognitive impairments in an adult Chinese population with T1DM and connection between cognitive dysfunction and DPN, which provides new insights on the pathogenic mechanisms of both cognitive impairment and DPN of T1DM patients. However, the definitive pathogenesis and alteration of cognitive impairment requires larger, prospective, longitudinal studies and appropriate, strict enrollment of subjects.

## Additional file

Additional file 1: The cut off points for NCV measures of peripheral nerves. (DOCX 22 kb)

## Abbreviations

BMI: Body mass index
CI: Confidence interval
CNS: Central nervous system
DKA: Diabetic ketoacidosis
DPN: Diabetic peripheral neuropathy
DR: Diabetic retinopathy
FPG: Fasting plasma glucose
HDL: High-density lipoprotein
LDL: Low-density lipoprotein
MCV: Motor nerve conduction velocity
MMSE: Mini-mental State Examination
MoCA: Montreal Cognitive Assessment
MRI: Magnetic resonance imaging
NCV: Nerve conduction velocity
OR: Odds ratio
SCV: Sensory nerve conduction velocity
T1DM: Type 1 diabetic mellitus
TC: Total cholesterol
TG: Triglyceride
WMH: White matter hyperintensity
