# Supplementary material for: Type 1 diabetes-associated cognitive impairment and diabetic peripheral neuropathy in Chinese adults: results from a prospective cross-sectional study
Source: BMC Endocr Disord. 2019 Mar 27;19:34. doi: 10.1186/s12902-019-0359-2 (PMC6437981; doi:10.1186/s12902-019-0359-2)
Supplement: Supplementary file 1 — The cut off points for NCV measures of peripheral nerves. (DOCX 22 kb) [file 12902_2019_359_MOESM1_ESM.docx]

**Table 1. The cut off points for NCV measures of median nerves**

| Age | SCV(sensory nerve conduction velocity) | | | | MCV(motor nerve conduction velocity) | | | |
| --- | --- | --- | --- | --- | --- | --- | --- | --- |
|  | Conduction velocity (m/s) | | Amplitude (uv) | | Latent period (ms) | | Amplitude (mv) | |
|  | Average | -20% | Average | Lower limit | Average | +20% | Average | Lower limit |
| 15-24 | 60.6 | 48.4 | 69.9 | 24.7 | 3.0 | 3.7 | 22 | 9 |
| 25-34 | 58.4 | 46.7 | 69.1 | 21.0 | 3.1 | 3.8 | 20 | 9 |
| 35-44 | 56.4 | 45.1 | 49.9 | 17.7 | 3.2 | 3.9 | 19 | 8 |
| 45-54 | 54.3 | 43.4 | 42.2 | 15.0 | 3.3 | 4.0 | 17 | 7 |
| 55-64 | 52.3 | 41.3 | 35.7 | 12.7 | 3.4 | 4.1 | 16 | 7 |
| 65-74 | 50.2 | 40.2 | 30.2 | 10.7 | 3.5 | 4.2 | 15 | 6 |
| 75-84 | 48.2 | 38.6 | 25.5 | 9.1 | 3.6 | 4.3 | 14 | 6 |

**Table 2. The cut off points for NCV measures of cubital nerves**

| Age | SCV | | | | MCV | | | |
| --- | --- | --- | --- | --- | --- | --- | --- | --- |
|  | Conduction velocity (m/s) | | Amplitude (uv) | | Latent period (ms) | | Amplitude (mv) | |
|  | Average | -20% | Average | Lower limit | Average | +20% | Average | Lower limit |
| 15-24 | 59.2 | 47.4 | 19.6 | 7.2 | 2.4 | 2.9 | 19 | 8 |
| 25-34 | 58.2 | 46.6 | 19.4 | 7.1 | 2.4 | 3.0 | 19 | 8 |
| 35-44 | 57.2 | 45.8 | 19.2 | 7.1 | 2.5 | 3.1 | 18 | 8 |
| 45-54 | 56.2 | 45.0 | 19.0 | 7.0 | 2.6 | 3.1 | 18 | 7 |
| 55-64 | 55.2 | 44.2 | 18.8 | 6.9 | 2.6 | 3.2 | 17 | 7 |
| 65-74 | 54.3 | 43.4 | 18.6 | 6.8 | 2.7 | 3.2 | 17 | 7 |
| 75-84 | 53.6 | 42.9 | 18.4 | 6.8 | 2.7 | 3.3 | 17 | 7 |

**Table 3. The cut off points for NCV measures of sural nerves**

| Age | SCV | | | |
| --- | --- | --- | --- | --- |
|  | Conduction velocity (m/s) | | Amplitude (uv) | |
|  | Average | -20% | Average | Lower limit |
| 15-24 | 57.8 | 46.2 | 15.6 | 4.3 |
| 25-34 | 57.6 | 46.1 | 12.2 | 3.3 |
| 35-44 | 57.3 | 45.8 | 9.5 | 2.6 |
| 45-54 | 57.0 | 45.6 | 7.4 | 2.0 |
| 55-64 | 56.8 | 45.4 | 5.8 | 1.6 |
| 65-74 | 56.5 | 45.2 | 4.5 | 1.2 |
| 75-84 | 56.3 | 45.0 | 3.5 | 1.0 |

**Table 4. The cut off points for NCV measures of common peroneal nerves**

| Age | SCV | | | | MCV | | | |
| --- | --- | --- | --- | --- | --- | --- | --- | --- |
|  | Conduction velocity (m/s) | | Amplitude (uv) | | Latent period (ms) | | Amplitude (mv) | |
|  | Average | -20% | Average | Lower limit | Average | +20% | Average | Lower limit |
| 15-24 | 60.9 | 48.7 | 4.5 | 0.9 | 2.8 | 3.6 | 15 | 6 |
| 25-34 | 59.7 | 47.8 | 4.0 | 0.8 | 2.9 | 3.7 | 15 | 6 |
| 35-44 | 58.4 | 46.7 | 3.5 | 0.7 | 3.0 | 3.7 | 15 | 6 |
| 45-54 | 57.2 | 45.2 | 3.1 | 0.6 | 3.0 | 3.8 | 15 | 6 |
| 55-64 | 55.9 | 44.7 | 2.7 | 0.6 | 3.1 | 3.9 | 15 | 6 |
| 65-74 | 54.6 | 43.7 | 2.5 | 0.5 | 3.2 | 3.9 | 15 | 6 |
| 75-84 | 53.4 | 42.7 | 2.2 | 0.4 |  |  |  |  |

**Table 5. The cut off points for NCV measures of posterior tibial nerves**

| Age | SCV | | | | MCV | | | |
| --- | --- | --- | --- | --- | --- | --- | --- | --- |
|  | Conduction velocity (m/s) | | Amplitude (uv) | | Latent period (ms) | | Amplitude (mv) | |
|  | Average | -20% | Average | Lower limit | Average | +20% | Average | Lower limit |
| 15-24 |  |  | 4.0 | 0.9 |  | 4.8 | 19 | 6 |
| 25-34 |  |  | 3.3 | 0.8 |  |  |  |  |
| 35-44 |  |  | 2.8 | 0.7 |  | 5.1 | 13 | 4 |
| 45-54 | 43.9 | 35.1 | 2.3 | 0.5 | 3.9 | 5.1 | 13 | 4 |
| 55-64 |  |  | 1.9 | 0.4 |  | 5.1 | 13 | 4 |
| 65-74 |  |  | 1.6 | 0.4 |  | 5.1 | 13 | 4 |
| 75-84 |  |  | 1.3 | 0.3 |  |  |  |  |
